# Preoperative high-intensity strength training combined with balance training can improve early outcomes after total knee arthroplasty

**DOI:** 10.1186/s13018-023-04197-3

**Published:** 2023-09-15

**Authors:** Jian-ning Sun, Yu-zhou Shan, Li-xia Wu, Ning Li, Fei-hu Xu, Xiang-ru Kong, Bei Zhang

**Affiliations:** 1 Department of Orthopedics, Nanjing Drum Tower Hospital Group Suqian Hospital, Suqian, China; 2 Department of Orthopedics, Suqian hospital affiliated to Xuzhou Medical University, Suqian, China; 3 Research Center, The Fourth Hospital of Hebei Medical University, Shijiazhuang, China

**Keywords:** Strength training, Postural balance, Knee osteoarthritis, Total knee arthroplasty

## Abstract

**Background:**

To investigate the effect of preoperative high-intensity strength training combined with balance training on the knee function of end-stage knee osteoarthritis (KOA) patients after total knee arthroplasty (TKA).

**Methods:**

A prospective study was conducted on end-stage KOA patients awaiting TKA. The patients were divided into an experimental group and a control group according to whether they received a preoperative training intervention. The differences in knee flexor–extensor strength, knee range of motion (ROM), timed up and go (TUG) test result, stair ascend/descend test result, Knee Society score (KSS) and Berg balance scale (BBS) score were assessed in both groups at baseline (T1), before operation (T2), 3 months after operation (T3), and 1 year after operation (T4).

**Results:**

After high-intensity strength training and balance training, the knee flexor–extensor strength, TUG test result, stair ascend/descend test result, and KSS were all significantly improved at T2 in the experimental group over the control group. At T3, the knee ROM, knee flexor–extensor strength, TUG test result, BBS score, and KSS clinical and functional scores were all significantly superior in the experimental group. The experimental group enjoyed a superiority in KSS clinical and functional scores until T4. Group × time and between-group interactions were found in all assessment indicators in both groups (*p* < 0.01).

**Conclusion:**

Preoperative high-intensity strength training combined with balance training can enhance the knee flexor–extensor strength and balance of patients with end-stage KOA in the short term and help improve early outcomes after KOA.

*Trial registration* ChiCTR2000032857, 2020-05-13.

## Introduction

Knee arthroplasty is an effective treatment for end-stage knee osteoarthritis (KOA), but 20–30% of patients still think that this procedure fails to meet their psychological expectations [1, 2]. The outcome of knee arthroplasty is associated with many factors, of which early postoperative functional recovery is considered a key one. Most scholars believe that early postoperative functional recovery is beneficial to the final function of joint and helps boost patients' confidence in recovery. Many studies have therefore focussed on accelerating early recovery.

As splitting of the quadriceps and patellar tendons or release of the peri-knee ligaments is required in total knee arthroplasty (TKA), early functional recovery is primarily hindered by surgical trauma-induced pain and decline in muscle strength. Multimodal analgesia has proven effective at controlling postoperative pain. Although patients feel that pain is no longer a major factor disrupting their exercise, the effect and speed of joint functional recovery remain unsatisfactory.

Muscle strength and activity decline by 60% and 17%, respectively, early after TKA, and the risk of decline becomes higher as muscle mass decreases with age [3]. Weakness in joint muscle strength following TKA is thought to be one of the main causes of postoperative functional decline. Preoperative knee flexor–extensor strength is considered a valid predictor for the joint function 1 and 2 years after TKA [4, 5]. Preoperative peri-knee muscle training is a potentially useful way to improve postoperative knee muscle strength and joint function [6–8]. On the other hand, there is an abundance of proprioceptors in the tendons, joint capsule, and meniscus around the knee joint, and the central nervous system adjusts the body's posture and maintains the balance of movement by receiving signals of movement, position, and vibration transmitted from the proprioceptors [9]. Patients with osteoarthritis of the knee suffer from decreased balance and knee instability due to proprioceptor damage, especially after undergone total knee replacement surgery [10]. Decreased joint position perception and reduced balancing ability are major consequences of KOA, both of which are thought to contribute to early postoperative falls and may impair the patient's activities of daily living. Balance training can help with hip contraction, abduction, standing, and knee lifting exercises.

According to a large body of literature, however, the joint function does not improve after these exercises, and the intensity of training engaged in by the patients varies, with even less attention paid to the degree of improvement in knee strength [11–13]. It is believed that balance training is as important as muscle training, as patients need to adapt to the altered kinematics caused by the joint prosthesis when getting out of bed or during daily activities [14, 15].

This study sought to improve the patient's preoperative knee strength and balance as much as possible through short-term, intense training. Whether early outcomes after TKA can be improved by preoperative high-intensity strength training combined with balance training was evaluated in this study. This study investigated the effect of short-term preoperative improvements in muscle strength and balance on the outcome after TKA.

## Subjects and methods

### General data

Patients diagnosed with unilateral KOA in the outpatient clinic and awaiting TKA in 2020–2021 were divided into an experimental group and a control group using a random number table. All procedures described in this study were reviewed by the Chinese Clinical Trial Registry (ChiCTR2000032857), were approved by the Hospital Ethics Committee (Ethics Number: 2022016), and complied with the requirements listed in the 1975 Declaration of Helsinki and its amendment in 2008.

Inclusion criteria: (1) Patients diagnosed with unilateral KOA and awaiting TKA, (2) those aged > 55 years, (3) those with clear consciousness and ability to communicate, and (4) those who provided consent to participate in this study.

Exclusion criteria: (1) Patients with a history of hip or knee surgery, (2) those with contraindications to exercise or diseases affecting leg activity, and (3) those who had incomplete follow-up data or withdrew from the study midway.

### Methods

Patients were instructed to train preoperatively for 4 weeks at home through an online video training and supervision module to enhance their cooperation and reduce time and medical costs. Similar methods have been found to be effective in previous studies [3, 16]. To improve muscle strength and balance within 4 weeks, further careful training programmes and experimental design are required, and training safety is particularly important, especially for elderly patients, who may have multiple underlying diseases.

Routine nursing and rehabilitation procedures were done in the control group. After admission, nurses conducted routine nursing education. Before operation, patients and their families attended a meeting jointly held by orthopaedic surgeons, physiotherapists, and anaesthetists on the operation method, the risks from the operation and anaesthetic, and postoperative rehabilitation. After operation, patients underwent active and continuous passive motion exercises to enhance their leg strength and increase knee mobility. These patients were followed up by telephone and clinic visit after discharge.

In the experimental group, 4-weeks preoperative strength training and balance training were additionally given, with the preoperative training programme and content kept confidential. One training session was required before the formal preoperative training, and the patients were tested for 1–4 days after mastering the training content and methods until they and the assessor were satisfied with their mastery of the training methods. The training programme aimed to enhance both strength and balance in the peri-knee muscles. The strength training focussed in particular on knee flexor–extensor strength, and weight on the knee gradually increased from 0 to a maximum of 2 or 3 kg. Strength training was followed by manual therapy, proprioceptive training, and ice compression. The detailed programme is shown in Table 1. Training sessions were completed within 4 weeks, 5 days per week (1 h per session). The physiotherapist responsible for this rehabilitation programme did not participate in any of the assessments during this study.

**Table 1:** Strength training programme for the experimental group

Warm-up exercises	Strength exercises	Balancing exercises	Ending exercise
1. Step up and down 20 times * 2	1. Knee flexion and extension 10RM*5	1. Stand with feet aligned for 1 min * 2	Light static stretching of hip abductors, knee flexors and ankle plantar flexor extensors for 5 min
2. Calf raises 20 times * 2		2. Standing on the forefoot and opposite heel for 1 min * 2	
3. Light resistance exercises 10 times	2. Hip flexion and extension. Adduction, abduction 10RM*5	3. Standing on the forefoot for 3 s * 15	
		4. Walk along a straight line10 m * 4	

Warm-up exercises were required before training to reduce muscle strain. Specifically, the strength training consisted of five sets of 10 repetitions, with a 60-s rest period between two sets. The intensity was regulated by weight and was based on the 10-repetition maximum. Exercises included knee extension and flexion; hip extension, adduction, and abduction; and standing balance. The training ended with a 5-min light static stretching of the hip abductors, knee flexors, and ankle plantar flexors and extensors.

With the same standardised preoperative protocol and surgical technique, TKA was performed by the same experienced surgeon on all patients. Posterior cruciate substituting prostheses and tourniquets were used in all cases.

### Assessment methods

The baseline data of patients in both groups were measured at the time of consent to participate in this study (T1), before operation (T2), 3 months after operation (T3), and 12 months after operation (T4). All assessors were professionally trained and were unaware of the grouping.

#### Muscle strength test

To measure the maximal isometric knee flexion and extension strength, the patients were instructed to sit on the edge of the examining table with their thighs in contact with the table, their hips at a constant angle (90°), and their trunk not tilted backwards. In an exercise before the formal assessment, the patients were asked to generate the maximum possible force on the dynamometer held by the assessor. In the isometric knee extension strength test, the dynamometer was fixed perpendicularly to the tibia by a strap 3 cm above the ankle joint, with the other end of the strap fixed to the base. In the isometric knee flexion strength test, the dynamometer was placed on the posterior part of the lower leg and fixed by a strap to the handle of a glass suction cup on the wall. The patients were told to perform three isometric maximal voluntary contractions, the average maximal strength of which was taken for analysis. The hand-held dynamometer showed good inter- and intra-assessor reliability in the assessment of knee flexors (ICC: 0.76–0.94) and knee extensors (ICC: 0.92–0.97) in the affected and unaffected knees [17].

#### Active knee range of motion (ROM)

The active knee ROM was measured using a digital goniometer. Specifically, the patients were instructed to lie in the prone position, with the knee extended, the hip in a neutral position, and the upper thigh exposed so that the greater trochanter could be seen. They flexed and extended the knee to its maximum, and the central pivot point of the goniometer was placed on the lateral epicondyle of the femur, with the proximal arm aligned with the lateral midline of the femur (the greater trochanter as a reference) and the distal arm aligned with the lateral midline of the fibula (the lateral malleolus and fibular head as references). In this way, the flexion and extension ROM was measured three times, and the average was taken. The knee ROM assessment of KOA patients showed high reliability, with ICCs of 0.96 and 0.81, respectively, for flexion and extension [18].

#### Stair ascend/descend test

The patients walked up and down four flights of stairs (50 cm wide, 15 cm high and 25 cm deep each flight) once as quickly but as safely as possible. They stood at the bottom of the first flight of stairs, went up the stairs, turned around on the top step, and kept going until both feet were on the floor. The handrail could be held if needed [19]. The total duration of this test was measured and averaged with a stopwatch, which stopped when the patient reached the starting line after going up and down the stairs [20]. The test was performed twice with a 30-s rest period. The purpose of the stair ascend/descend test is to assess lower limb muscular strength and endurance, and it is generally accepted that the shorter the time taken to complete the task, the higher the tester's level of lower limb muscular strength and endurance [21]. The stair ascend/descend test was highly reliable, with a test–retest reliability coefficient of 0.93 and an ICC of 0.94 for inter-assessor reliability [22].

#### Timed up and go (TUG) test

The TUG test is an easy-to-perform test that assesses mobility, leg function, and fall risk without any specific equipment needed [23]. The patients stood up from a standard armchair (without using their arms), walked to a straight line on the floor 3 m away, turned around, walked back to the chair, and sat down again. The duration of the above process was recorded as the TUG result [24].

#### Knee function

The Knee Society score (KSS) is one of the most widely used knee function scores in clinical practice. The Knee Score consists of a clinical score and a functional score. The total score is 100 points, with higher scores meaning better knee function [25]. KSS assesses knee pain, ROM, and stability and serves as a reliable indicator of knee recovery and function.

#### Balance

The Berg balance scale (BBS) was used to assess the patient's balance. The patient does 14 related activities, such as sit-to-stand transfer, unsupported standing, and unsupported sitting. The maximum BBS score is 56. The higher the score, the better the balance.

### Statistical analysis

The KSS score was defined as the primary outcome variable for the power analysis. In the pre-test, the standard deviation and error of the baseline KSS were 7.5 and 2.9, respectively, the statistical power was 90%, and the level of significance was *p* < 0.05. At least 29 patients were needed in each group.

IBM SPSS 21.0 software (Chicago, USA) was used for statistical analysis. Measurement data such as age and joint function scores are described as mean ± standard deviation ( $\overline{x}$ ± s), and data such as osteoarthritis *K*–*L* classification and sex are described as percentage (%). Data were tested for normality using the Shapiro–Wilk test. Student’s *t*-test was used to compare the means of normally distributed data between the two groups, the Mann–Whitney test for non-normally distributed data, and the chi-squared test for differences in composition ratios. Differences in ROM, TUG test result, muscle strength test result, KSS and BBS scores were analysed using repeated-measures analysis of variance, where group, time, and group × time interaction were the independent variables. *p* < 0.05 was significant.

## Results

**Fig. 1:**
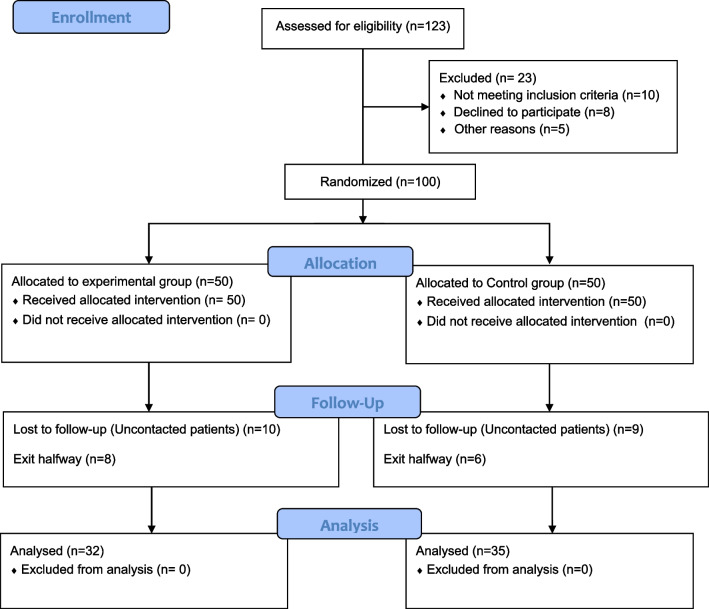
Study flowchart

A total of 100 patients were included, 50 in each group. Thirty-three patients withdrew from the study midway, including 18 in the experimental group and 15 in the control group. Finally, 67 patients were assessed (Fig. 1). The experimental group consisted of 32 patients, including nine males and 23 females aged 66.4 years on average. There were 35 patients in the control group, including 12 males and 23 females aged 68.5 years (Table 2).

**Table 2:** The demographic characteristics of the patients in the experimental group and the control group

	Experimental group (*N* = 32)	Control group (*N* = 35)	*p*
Age (year)	66.4 ± 8.3	68.5 ± 7.9	0.844
Gender (male/female)	9/23	12/23	0.587
BMI (kg/m^2^)	22.6 ± 3.3	23.6 ± 2.5	0.489
Surgical side (left/right)	17/15	16/19	0.544
Kellgren–Lawrence grade (3/4)	25/7	32/3	0.127

Significant between-group interactions over time were found for all indicators (Tables 3 and 4). At T1, no significant differences were observed in any indicator in either group. At T2, the knee flexor–extensor strength in the experimental group increased by 6.0 kg and 4.9 kg, respectively, over that at T1, which improvement was greater than the control group’s (14.2 ± 3.9 vs. 9.0 ± 0.9, *p* < 0.001; 24.8 ± 2.5 vs. 21.3 ± 2.3, *p* < 0.001). The better knee flexor–extensor strength in the experimental group was maintained at T2 and T3 (respectively: 10.4 ± 1.6 vs. 9.1 ± 0.7, *p* < 0.001; 21.9 ± 8.0 vs. 16.3 ± 5.8, *p* = 0.002).

**Table 3:** Scores for all physical measures

Variable	Testing time	Mean (95%CI)		*p* value (Group * time)	*p* value (Between-group difference)
		Experimental group	Control group		
Knee ROM (°)	T1	92.3 (86.8–97.9)	89.1 (82.8–95.4)	< 0.001*	0.443
	T2	95.8 (88.2–103.4)	92.6 (85.5–99.7)		0.529
	T3	114.1 (109.0–119.2)	104.8 (97.2–112.3)		0.045*
	T4	124.2 (120.0–128.4)	123.2 (119.0–127.4)		0.739
Isometric knee flexion (kg)	T1	8.2 (7.8–8.6)	8.5 (8.1–8.9)	< 0.001*	0.229
	T2	14.2 (12.7–15.6)	9.0 (8.7–9.3)		< 0.001*
	T3	10.4 (9.9–11.0)	9.1 (8.9–9.4)		< 0.001*
	T4	14.6 (13.2–16.0)	15.3 (14.1–16.5)		0.444
Isometric knee extension (kg)	T1	19.9 (18.5–21.4)	21.7 (20.1–23.4)	< 0.001*	0.102
	T2	24.8 (23.9–25.7)	21.3 (20.5–22.1)		< 0.001*
	T3	21.9 (19.0–24.8)	16.3 (14.3–18.3)		0.002*
	T4	25.1 (23.8–26.4)	25.5 (24.6–26.4)		0.581
Stair test (s)	T1	8.9 (8.7–9.0)	8.8 (8.7–8.9)	< 0.001*	0.696
	T2	7.5 (6.7–8.4)	8.7 (8.5–8.8)		0.010*
	T3	8.8 (8.4–9.2)	9.4 (8.9–9.8)		0.052
	T4	7.8 (7.1–8.5)	8.3 (7.5–9.0)		0.340
Timed up and go (s)	T1	9.0 (8.7–9.2)	9.4 (8.9–9.8)	< 0.001*	0.100
	T2	7.8 (7.1–8.6)	9.3 (8.9–9.7)		0.001*
	T3	8.1 (7.1–9.0)	9.1 (8.8–9.3)		0.028*
	T4	7.4 (6.4–8.3)	8.1 (7.2–9.0)		0.243

*ROM*: Range of motion. T1-4: The time of consent to participate in this study (T1), before operation (T2), 3 months after operation (T3), and 12 months after operation (T4)

*Denotes significant difference

**Table 4:** Scores for all the questionnaires

Variable	Testing time	Mean (95%CI)		*p* value (Group*time)	*p* value (Between-group difference)
		Experimental group	Control group		
KSS function score	T1	42.5 (40.4–44.6)	42.7 (40.7–44.8)	< 0.001*	0.883
	T2	46.8 (44.2–49.3)	43.4 (41.4–45.5)		0.045*
	T3	84.0 (80.6–87.4)	79.6 (77.5–81.6)		0.023*
	T4	92.8 (89.6–96.0)	87.9 (84.3–91.4)		0.043*
KSS clinical score	T1	49.2 (45.9–52.5)	50.3 (46.0–54.6)	< 0.001*	0.684
	T2	54.4 (50.0–58.7)	50.7 (46.6–54.8)		0.211
	T3	84.7 (82.5–86.8)	80.6 (77.6–83.7)		0.034*
	T4	93.7 (90.7–96.6)	88.6 (85.3–92.0)		0.027*
BBS	T1	40.2 (36.9–43.1)	39.8 (36.9–42.8)	< 0.001*	0.281
	T2	41.6 (34.8–39.1)	38.5 (35.2–41.8)		0.139
	T3	43.7 (41.2–46.2)	35.7 (32.1–39.3)		0.001*
	T4	50.5 (48.8–52.2)	51.3 (49.2–53.3)		0.571

KSS: Knee Society score. BBS: Berg balance scale. T1-4: The time of consent to participate in this study (T1), before operation (T2), 3 months after operation (T3), and 12 months after operation (T4)

*Denotes significant difference

At T2, the stair ascend/descend test and TUG test results were significantly better in the experimental group than the control group [(7.5 ± 2.4) s vs. (8.7 ± 1.4) s, *p* = 0.010; (7.8 ± 2.1) s vs. (9.3 ± 1.1) s, *p* = 0.001]. At T3, the knee ROM and TUG test result were significantly better in the experimental group [(114.1 ± 14.1)4 vs. (104.8 ± 22.0)2, *p* = 0.045; (8.1 ± 2.6) s vs. (9.1 ± 0.7) s, *p* = 0.028].

At T2, the KSS functional score in the experimental group was better than that in the control group (46.8 ± 7.4 vs. 43.4 ± 5.7, *p* = 0.045). At T3, the KSS clinical and functional scores in the experimental group showed a greater improvement than those in the control group (84.0 ± 9.4 vs. 79.6 ± 6.0, *p* = 0.023; 84.7 ± 5.9 vs. 80.6 ± 8.9, *p* = 0.034). The experimental group enjoyed the superiority in KSS clinical and functional scores until at least T4 (92.8 ± 8.9 vs. 87.9 ± 10.5, *p* = 0.043; 93.7 ± 8.2 vs. 88.6 ± 9.8, *p* = 0.027).

At T3, the experimental group showed better balance and BBS score (43.7 ± 6.9 vs. 35.7 ± 10.5, *p* = 0.001), but the benefits were not maintained at T4 (50.5 ± 4.7 vs. 51.3 ± 6.0, *p* = 0.571).

## Discussion

The main finding of this study was that compared with the control, high-intensity strength training combined with balance training in the experimental group significantly ameliorated knee ROM, flexor–extensor strength and balance and achieved better postoperative joint function.

The goal of this study was to enhance the patient's perioperative knee muscle strength through high-intensity strength training, which was achieved. At T2, the knee flexor–extensor strength in the experimental group increased by 6.0 kg and 4.9 kg, respectively, compared with T1, whereas it almost had no changes in the control group, suggesting that preoperative high-intensity strength training can enhance knee flexor–extensor strength in the short term and that the benefit can last until 3 months after operation. The knee extensors are damaged due to the splitting of the quadriceps and patellar tendons during TKA, and the patient should adapt to the knee prosthesis, so knee flexor–extensor strength declines after operation. Even so, the muscle strength was better in the experimental group than in the control group, which benefitted the knee functional recovery. Moreover, the mean knee ROM in the experimental group was 9.3° greater than that in the control group at 3 months after operation, which was significantly influenced by the peri-knee muscle strength. Similarly, Matassi et al. [26] investigated 125 patients preparing for TKA, with no additional intervention in the control group, while 61 subjects in the experimental group received a 6-week home exercise program prior to TKA, and the preoperative home exercise program focused on lower extremity muscle strength and soft tissue flexibility. He found that the knee ROM reached 90° at 5.8 days after operation in their experimental group but at 6.9 days in their control group. Joaquin et al. [3] evaluated the effectiveness of a high-intensity preoperative resistance training program for patients awaiting TKA. The training program lasts 8 weeks, 3 times a week, and consists mainly of warm-up training, main procedure training(10 repetitions of resistance training per set with 60 s intervals at an intensity of 10 RM), and relaxation training at the end of each session. He concluded that postoperative knee flexor strength was significantly better in their experimental group receiving preoperative training than it was in their control group, but their patients had difficulty improving knee extensor strength due to the damage to the knee extensor.

The high-intensity strength training persistently benefited knee flexor–extensor strength in the experimental group over time, so their KSS clinical and functional scores were always higher than the control group’s. The ability to go up and down stairs and to walk is a major manifestation of the joint mobility and function as part of a person’s activities of daily living [27, 28]. DM et al. [11] showed no significant improvement was made in the stair ascend/descend test or TUG test results in patients awaiting TKA after additional progressive strength training 2–3 times a week for 6 consecutive weeks. Similarly, the same results were found in the study of Jose et al. [29] who conducted a lower extremity muscle strength training program 3 times a week for 8 weeks. In this study, the stair ascend/descend test result in the experimental group at T2 was better than that in the control group, but the benefit was not maintained throughout the postoperative period. In contrast, the experimental group did enjoy superiority in the TUG test result until T3. The stair ascend/descend test and TUG test results had no significant differences at T4 between the two groups. It can be seen that improvement in knee flexor–extensor strength influenced the stair ascend/descend test result at T2, as knee flexors and extensors play an important roles in stair climbing. However, the knee extensor is the major player in TUG test, and it was damaged during operation and thus was more significantly affected. In the experimental group, preoperative training contributed to better results.

Significant group × time and between-group differences were found for all indicators. Patients in both groups had significant improvements in the KSS clinical and functional scores and BBS score as a result of the surgical intervention, but the surgical benefits were more obvious in the experimental group. It can be inferred that preoperative high-intensity strength training combined with balance training can help improve the outcome of TKA, which can synergize with other measures of improving joint function, such as modified surgical techniques and analgesia, to facilitate rehabilitation and reduce the patient's postoperative need for social and medical care.

Unlike previous studies, this study provided high-intensity strength training combined with balance training to enhance the postoperative joint function of patients in the short term. These were aimed at KOA patients who had been scheduled to undergo TKA. Choosing an appropriate intensity and duration of training are vital for enhancing muscle strength [30]. In many previous preoperative training sessions [11, 31], patient training intensity was designed to be moderately fatiguing instead of a perception of a maximal number of repetitions (i.e. RM) or a percentage of a maximum load (i.e. % 1RM). We adopted a training protocol of patient self-perceived intensity in order to emphasize the difference from previous conventional strength training. The advantage of such strength training is that individualized strength training can be achieved, both for strength training purposes and to reduce the risk of injury. A training intensity of 10RM is considered an effective means of improving muscle strength [3, 29, 32]. Patients who experience more pain during high-intensity training, especially those who avoid knee pain by significantly reducing their daily activities, have weaker muscle strength and need to be more careful and gentle in the early stage of training. Moreover, additional balance training during muscle strength training can significantly promote balance early postoperatively, and earlier functional exercise corresponds to better long-term outcomes, although a difference in balance was only observed at T3. With better balance, early postoperative rehabilitation exercise can be easier, and the risk of falls due to impaired proprioception can be reduced. The findings of this study demonstrate the effectiveness of preoperative high-intensity strength training combined with balance training, which may encourage more patients to undergo preoperative training to achieve better surgical outcomes.

This study had the following limitations: (1) the dosage of analgesics in the two groups was not recorded, so the difference in the dosage may have produced deviations in some results. The impact of this potential bias was not reduced by introducing VAS scores. (2) There were only four time points for measurement, which was too few to observe the variation in values in more detail. (3) The last measurement was performed at 12 months after operation, so the long-term knee function was not observed. Therefore, a longer follow-up period is required. (4) Kinesiophobia is one of the important potential factor affecting the results of the strength training. It needs to be taken into account in further studies. (5) The sample was too small to yield convincing conclusions, so large, multicentre studies are needed.

## Conclusion

Preoperative high-intensity strength training combined with balance training can enhance the knee flexor–extensor strength and the balance of patients with end-stage KOA in the short term, help improve the early postoperative knee function, and accelerate rehabilitation.

## Data Availability

All data generated or analyzed during this study are included in this published article.
